# Exogenous glutamate potentiates gentamicin to kill multidrug- and carbapenem-resistant *Pseudomonas aeruginosa* by activating the biosynthesis of unsaturated fatty acids

**DOI:** 10.1128/msystems.01234-25

**Published:** 2025-10-13

**Authors:** Hao-Feng Lai, Li Pan, Kang-Yu Song, Zhen-Yuan Dai, Ying Liang, Wu Yuan, Zhuang-Gui Chen, Li-Fen Yang

**Affiliations:** 1 Children’s Medical Center, The Third Affiliated Hospital, Sun Yat-sen University 26469 , Guangzhou, China; 2 Department of Allergy, The Third Affiliated Hospital, Sun Yat-sen University 26469 , Guangzhou, China; 3 Department of Biomedical Engineering, The Chinese University of Hong Kong, Hong Kong SAR, China; San Diego State University, San Diego, California, USA

**Keywords:** *Pseudomonas aeruginosa*, multidrug resistance, carbapenem resistance, glutamate, gentamicin, membrane permeability, isotope-tracing analysis

## Abstract

Multidrug- and carbapenem-resistant *Pseudomonas aeruginosa* (MDR-PA and CR-PA) are difficult to control due to the predicament caused by their limited membrane permeability. The metabolic reprogramming approach is an effective strategy to promote membrane permeability. In this study, a gas chromatography-mass spectrometer-based metabolomics identified decreased abundance of glutamate as the most characteristic feature in gentamicin-resistant *P. aeruginosa* (PA-R_GEN_). Exogenous glutamate enhanced gentamicin killing to lab-evolved PA-R_GEN_ as well as clinical MDR-PA and CR-PA isolates. By applying a multi-faceted approach, including glutamate-reprogramming metabolomics, isotope-tracing analysis, glutamate-reprogramming lipidomics, membrane permeability measurement, and oleic acid replacement test, we demonstrated that the glutamate metabolic flux increases the biosynthesis of unsaturated fatty acids and decreases the biosynthesis of saturated fatty acids. This change in lipid composition promotes membrane permeability and enhances gentamicin uptake in the presence of glutamate. However, the opposite phenotypes were exhibited in MDR- and CR-PA in the absence of glutamate. These results identify an effective reprogramming metabolite to combat MDR- and CR-PA with gentamicin and reveal a resistance mechanism of membrane permeability that limits drug uptake and its reversal approach in MDR- and CR-PA.

**IMPORTANCE:**

Antibiotic-resistant *Pseudomonas aeruginosa* is a major clinical challenge due to limited drug uptake. This study shows that exogenous glutamate restores gentamicin efficacy by reprogramming bacterial metabolism to enhance membrane permeability. The effect is mediated through increased biosynthesis of unsaturated fatty acids, which is further confirmed by oleic acid supplementation. These findings reveal a novel metabolic approach to overcome multidrug and carbapenem resistance, offering a promising adjunct strategy to improve antibiotic treatment outcomes.

## INTRODUCTION

*Pseudomonas aeruginosa* is a gram-negative opportunistic pathogen that causes acute or chronic infections in immunocompromised individuals with chronic obstructive pulmonary disease, cystic fibrosis, trauma, burns, sepsis, or ventilator-associated pneumonia (1). It utilizes high levels of intrinsic and acquired resistance mechanisms to counter most antibiotics. Thus, the emergence of multidrug-resistant (MDR) *P. aeruginosa* is a critical problem in clinical practice. Particularly, only 36% of the patients infected with carbapenem-resistant *P. aeruginosa* (CR-PA) survive without complications, whereas the remaining patients either die or experience at least one serious complication (2). In 2024, the WHO published a list of priority pathogens that includes CR-PA, which was given “high priority status” since it represents a great threat to humans (3). However, effective control approaches are still lacking and are being explored. The combination of β-lactams and β-lactamase inhibitor(s) is implemented as the main strategy to solve this problem, but it is undermined by the resistance to not only β-lactams but also β-lactamase inhibitor(s), as well as the diversity of β-lactamase (4). Therefore, we need to update our research ideas.

Recently developed metabolome/metabolic state-reprogramming is a promising way to combat antibiotic-resistant bacteria by repurposing existing antibiotics to which resistance has already developed, which has achieved great success in killing clinically isolated *Escherichia coli*, *Edwardsiella tarda*, and *Vibrio alginolyticus* (5–10). We established a glucose-potentiated amikacin-mediated approach that was effective against both lab-evolved cefoperazone/sulbactam-resistant and clinically multidrug-resistant *P. aeruginosa* (11). We also showed that the upregulation of fatty acid biosynthesis forms a characteristic feature of the metabolomes in ciprofloxacin-resistant *P. aeruginosa*, which provides a target pathway for combating ciprofloxacin-resistant *P. aeruginosa* (12). However, whether the metabolites-enabled killing is effective against CR-PA remains unknown.

Here, gas chromatography-mass spectrometer (GC-MS)-based metabolomics approach was used to characterize the differential metabolic profile of lab-evolved gentamicin-sensitive and -resistant *P. aeruginosa* ATCC27853 (PA-S and PA-R_GEN_, respectively). Suppressed glutamic acid was identified as the most crucial biomarker. Exogenous glutamic acid was able to potentiate gentamicin to kill not only lab-evolved PA-R_GEN_ and clinically multidrug-resistant *P. aeruginosa* but also CR-PA isolates. An underlying mechanism is attributed to the elevation of membrane permeability to increase drug uptake by regulating the biosynthesis of unsaturated fatty acids.

## RESULTS

### Metabolic profiles of PA-R_GEN_

**Fig 1:**
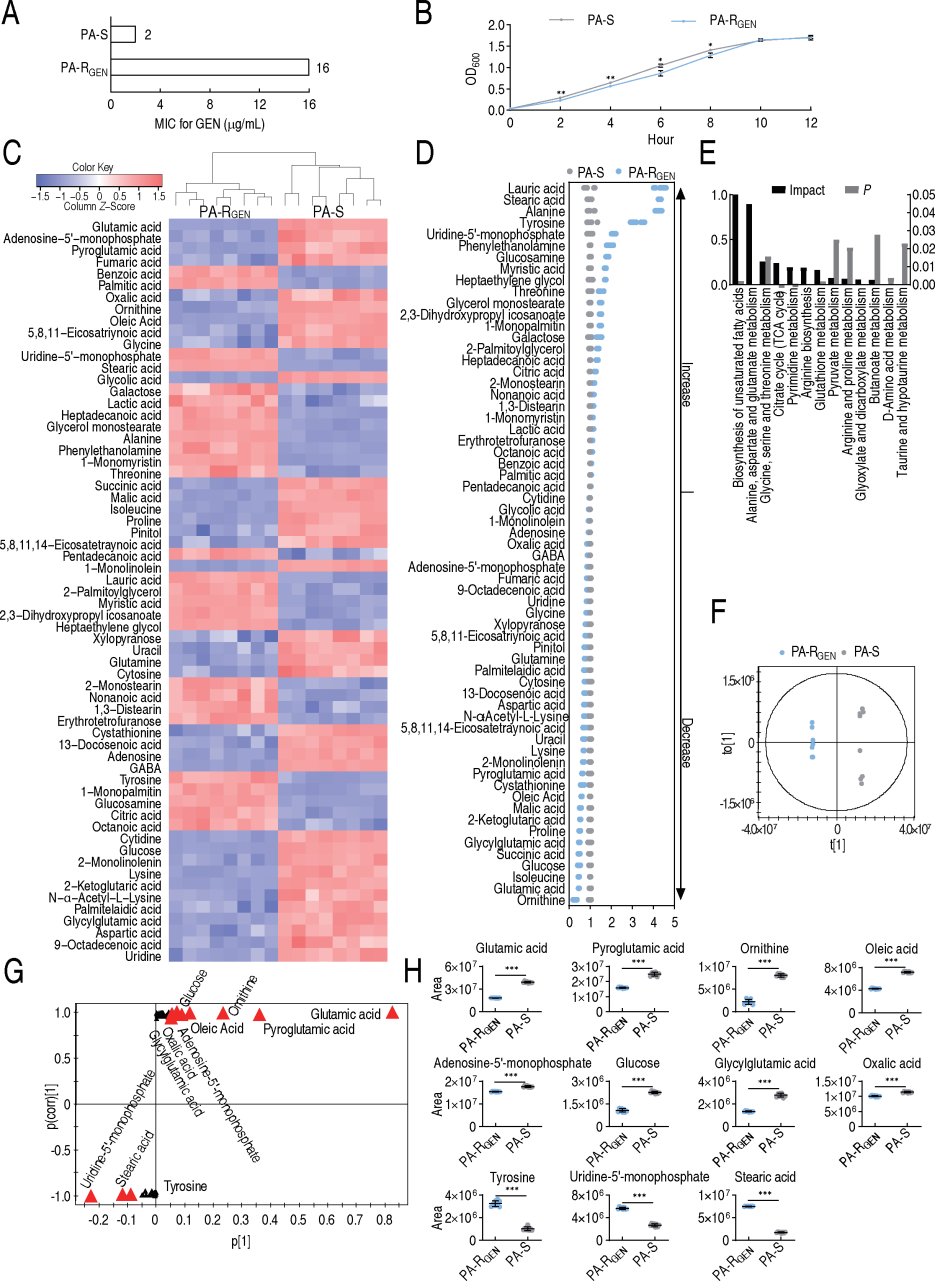
Metabolic profiles of PA-R_GEN_. (**A**) MIC for gentamicin of PA-R_GEN_ compared with PA-S in MH broth. (**B**) Growth curve of PA-R_GEN_ compared with PA-S in LB broth. (**C**) The heatmap of the abundance of 63 differential metabolites between PA-R_GEN_ and PA-S. The color change from blue to red represents a change in metabolite abundance from low to high, and the heat map scale is shown on the left top. (**D**) Fold change analysis of the 63 differential metabolites, corresponding to data in (**C**). (**E**) Enriched metabolic pathways (*P* < 0.05) of the 63 differential metabolites by the website analysis (https://www.metaboanalyst.ca/). (**F**) OPLS-DA of metabolomes between PA-R_GEN_ and PA-S. Each dot represents the biological and technical replicates analysis of samples in the plot. (**G**) S-plot generated from OPLS-DA. Predictive component p[1] and correlation p(corr)[1] differentiate PA-R_GEN_ and PA-S. Triangle represents an individual metabolite, where potential biomarkers are highlighted in red, which is greater than or equal to 0.05 and 0.5 for the absolute value of covariance p and correlation p(corr), respectively. Otherwise, the triangle is marked in black. (**H**) The scatter plot of potential biomarkers in (**G**). Except for (**H**), which is four biological replicates and two technical replicates, all other results are displayed as mean ± SEM of four biological replicates. * *P*＜0.05, ** *P*＜0.01, and *** *P*＜0.001.

To study the metabolic shifts in gentamicin resistance, the ATCC 27853 reference strain was serially passaged to generate resistant derivatives, enabling comparative metabolic analysis. To do this, the antibiotic-sensitive ATCC 27853 was sequentially cultured in LB broth with or without 1/2 MIC of gentamicin, leading to gentamicin-resistant *P. aeruginosa* with 8-fold MICs (PA-R_GEN_) and gentamicin-sensitive *P. aeruginosa* with unchanged MIC (PA-S), respectively (Fig. 1A). The growth curve showed that slower growth was detected in PA-R_GEN_ than PA-S during the 2−8 h period (Fig. 1B). Therefore, differential phenotypes are detected between PA-R_GEN_ and PA-S. A GC-MS-based metabolomic approach was used to characterize the metabolic profiles in PA-R_GEN_ and PA-S. Four biological samples with two technical replicates were used for each strain. After the removal of any known artificial peaks and the integration of the same compounds, 93 metabolites with reliable signals were identified per strain. The correlation coefficient between technical replicates was 0.9999 (Fig. S1A), demonstrating high reproducibility of the data. The metabolites were categorized into carbohydrates (15%), lipids (30%), amino acids (20%), nucleotides (8%), and others (27%) (Fig. S1B). Metabolic profiles of the two strains were visualized as a heatmap, where PA-R_GEN_ and PA-S were separated clearly (Fig. S1C).

A total of 63 of 93 metabolites showed significantly different abundances between PA-R_GEN_ and PA-S (*P*  <  0.01, two-sided Mann-Whitney *U* test), as visualized in the heatmap (Fig. 1C). The fold change value of a metabolite is calculated as the abundance of the metabolite in PA-R_GEN_ divided by its abundance in PA-S, ranging from 0.27 to 4.3. Among the 63 differential metabolites, 27 increased and 36 decreased in PA-R_GEN_ compared with PA-S. Notably, ornithine, glutamate, isoleucine, and glucose were among the most depleted metabolites in PA-R_GEN_ (Fig. 1D). The differential metabolites were classified into 16% carbohydrates, 39% lipids, 26% amino acids, 10% nucleotides, and 8% others (Fig. S1D). By inputting the 63 differential metabolites into MetaboAnalyst (13), a total of 13 enriched pathways were identified. According to the impact, the top two enriched metabolic pathways were the biosynthesis of unsaturated fatty acids and alanine, aspartate, and glutamate metabolism (Fig. 1E; Fig. S1E). Orthogonal partial least squares discriminant analysis (OPLS-DA) was adopted to recognize the sample patterns of metabolomes. Component t[1] separated PA-R_GEN_ from PA-S (Fig. 1F). Discriminating variables were identified by S-plot. In the plot of predictive correlation between p[1] and p(corr)[1], the red triangle indicates the differential metabolites that had more significant variability (covariance p and correlation p(corr) absolute values greater than or equal to 0.05 and 0.5, respectively). A total of 11 metabolites, including eight decreased metabolites (glutamic acid, pyroglutamic acid, ornithine, oleic acid, adenosine-5'-monophosphate, glucose, glycylglutamic acid, and oxalic acid) and three increased metabolites (tyrosine, uridine-5'-monophosphate, and stearic acid), were identified as biomarkers in PA-R_GEN_ (Fig. 1G). Their abundances are presented in scatter plots (Fig. 1H). Among them, glutamate is the most decreased biomarker (Fig. 1H), with glutamate being the second most decreased metabolite in the fold-change analysis (Fig. 1D). Therefore, these results suggest that the suppressed glutamate is a key feature that is responsible for the resistance.

### Glutamate alters the sensitivity of *P. aeruginosa* to gentamicin

**Fig 2:**
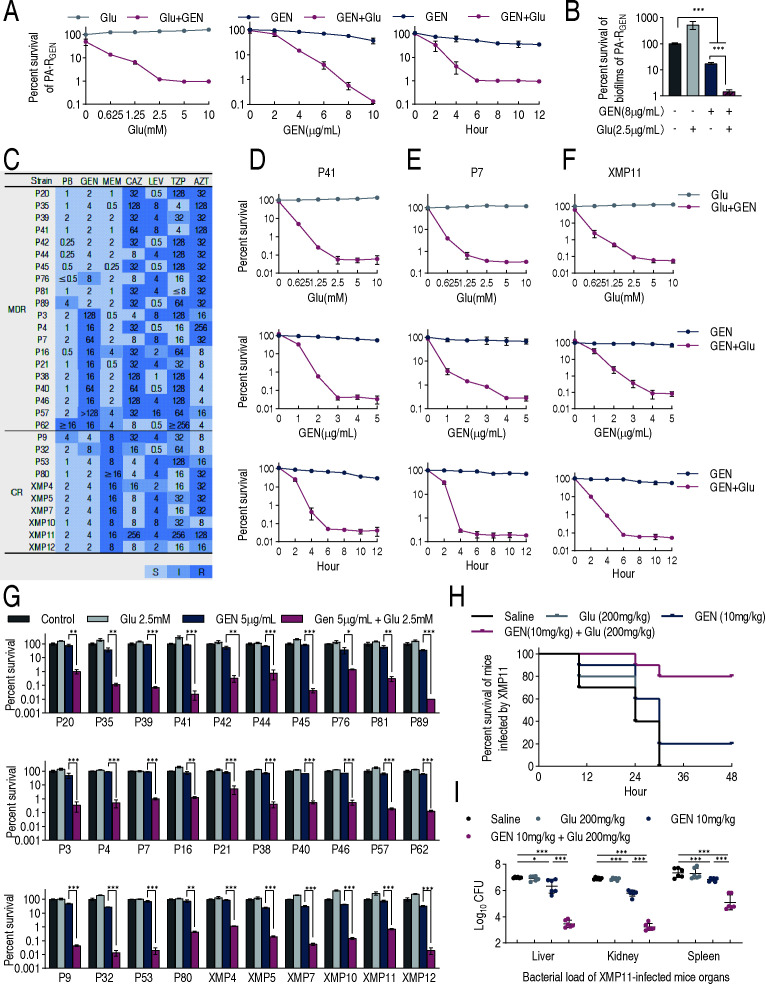
Glutamate alters the sensitivity of *P. aeruginosa* to gentamicin. (**A**) The effects of glutamate (Glu) concentration, gentamicin (GEN) dosage, and bactericidal time on the bactericidal effect when acting on PA-R_GEN_ in M9 medium. (**B**) The synergistic effect of glutamate and gentamicin on the eradication of biofilms of PA-R_GEN_ in M9 medium. (**C**) MICs of various antibiotics for clinical strains of *P. aeruginosa* in MH broth. (**D–F**) The effects of glutamate concentration, gentamicin dosage, and bactericidal time on the bactericidal effect when acting on P41, P7, and XMP11 in M9 medium, respectively. (**G**) The synergistic effect of glutamate and gentamicin on the strains in (**C**). (**H**) Percent survival of mice in the absence or presence of 10 mg/kg gentamicin and/or the indicated dose of 200 mg/kg glutamate. Mice were intraperitoneally infected with 1.47 × 10^7^ CFU of XMP11 and divided into four groups to be treated by intramuscular injection as indicated post 1 h after infection. There were 10 mice in each group. Survival of mice was monitored for 48 h. (**I**) The synergistic effect of glutamate and gentamicin in reducing the bacterial load of CR-PA-infected mice. The results are displayed as means ± SEM from six biological replicates. * *P*＜0.05, ** *P*＜0.01, and *** *P*＜0.001. Unless otherwise stated, the results are displayed as mean ± SEM of three biological replicates. * *P*＜0.05, ** *P*＜0.01, and *** *P*＜0.001.

To explore the potential of glutamate to modulate antibiotic susceptibility in *P. aeruginosa*, we assessed the survival of PA-R_GEN_ and XMP11 in M9 medium supplemented with or without glutamate across 22 antibiotics representing nine classes: aminoglycosides, β-lactams, carbapenems, macrolides, quinolones, polymyxins, tetracyclines, penicillins, and lincosamides. XMP11 is a carbapenem-resistant strain of *P. aeruginosa* that also exhibits a classic multidrug-resistant phenotype. Glutamate demonstrated varying levels of synergistic activity with most antibiotics against both PA-R_GEN_ and XMP11 strains, with the exception of polymyxins and lincosamides. Notably, the strongest potentiation effects were observed with aminoglycosides, particularly gentamicin and kanamycin (Fig. S2A). Given its superior potentiation by glutamate, gentamicin was chosen for further study. Glutamate potentiated the bactericidal effect of gentamicin on PA-R_GEN_ in a glutamate dose- and time-dependent manner, up to 2.5 mM and 6 h, after which the effect plateaued (Fig. 2A). Glutamate also promoted the killing with increasing gentamicin dose (Fig. 2A). Glutamate-potentiated gentamicin killing was effective against PA-R_GEN_ biofilms, with an elevation by 12.34-fold (Fig. 2B). Additionally, glutamate was substituted with ornithine, isoleucine, or glucose to assess their potential to enhance gentamicin-mediated bacterial killing. Although the other three metabolites exhibited some potentiation of gentamicin activity, their bactericidal effects were weaker than that of glutamate (Fig. S2B), indicating that glutamate remains the optimal choice. It is interesting to know whether glutamate potentiates gentamicin killing against clinical *P. aeruginosa* isolates. For this purpose, 30 isolates were collected. They were classified into 20 MDR-PA strains (resistant to at least three classes of antibiotics) and 10 CR-PA strains (resistant to at least three classes of antibiotics and meropenem). Among 20 MDR-PA strains, 10 were sensitive to gentamicin and 10 were resistant (Fig. 2C). Three isolates, P41, P7, and XMP11, were selected as representatives from MDR-PA with sensitivity to gentamicin, MDR-PA with resistance to gentamicin, and CR-PA. The three representatives were used for the analysis of the killing conditions. Similar to lab-evolved PA-R_GEN_, the viability of P41, P7, and XMP11 reduced with increasing glutamate and gentamicin concentrations and longer incubation time, and the maximum killing was observed at 2.5 mM glutamine and 3–5 μg/mL gentamicin (P41, 5 µg/mL; P7, 3 µg/mL; XMP11, 4 µg/mL) during 6–12 h of treatment (Fig. 2D through F). All 30 isolates were treated in the absence or presence of 5 µg/mL gentamicin and/or 2.5 mM glutamate for 6 h. Glutamate enhanced gentamicin-mediated killing across all 30 strains, with varying degrees of synergy, with the most pronounced synergy culminating in an 8,296-fold increase in killing efficacy (Fig. 2G). Compared with the control without gentamicin and glutamate, glutamate alone displayed similar (almost) or a bit higher (a few) survival, and gentamicin alone exhibited low (most) and similar (a few) viability. However, lower survival was measured in the synergy of gentamicin and glutamate than in gentamicin alone (Fig. 2G). Since 5 µg/mL gentamicin exceeded the MIC (minimum inhibitory concentration) for most strains (Fig. 2C), a sub-MIC concentration gradient (<5 µg/mL) was employed to assess whether glutamate could potentiate the bactericidal activity of gentamicin at subinhibitory concentrations. The results demonstrate that glutamate’s potentiating effect was maintained even at sub-MIC levels (Fig. S2C).

Furthermore, the synergy efficiency was evaluated in a mouse model with systemic infection caused by XMP11. Mice were infected and then treated 1 h later with saline, 200 mg/kg glutamate, 10 mg/kg gentamicin, and both (*n* = 10 per group). All mice in the saline-only and glutamate-only groups succumbed to infection, whereas 20% and 80% survivals were observed in the gentamicin group and glutamate + gentamicin group, respectively (Fig. 2H). Consistently, bacterial loads in the liver, kidney, and spleen were lower in the synergy of gentamicin and glutamate groups than in gentamicin alone (Fig. 2I).

### Efflux and influx kinetics of gentamicin in XMP11 in the presence or absence of glutamate

**Fig 3:**
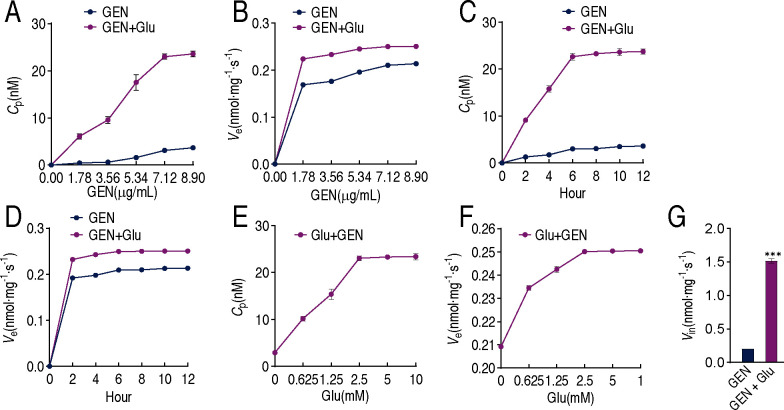
Efflux and influx kinetics of gentamicin in XMP11 in the presence or absence of glutamate. (**A and B**) Relationships of increasing extracellular gentamicin concentrations with intracellular gentamicin concentrations (*C*_p_) (**A**) and efflux rate (*V*_e_) (**B**) in the presence or absence of 2.5 mM glutamate and the indicated concentration of gentamicin. Note that 1.78, 3.56, 5.34, 7.12, and 8.90 µM gentamicin are equal to 1, 2, 3, 4, and 5 µg/mL gentamicin, respectively. (**C and D**) Relationships of increasing incubation periods with *C*_p_ (**C**) and *V*_e_ (**D**) in the presence of 7.12 µM gentamicin with or without 2.5 mM glutamate. (**E and F**) Relationships of increasing extracellular glutamate with *C*_p_ (**E**) and *V*_e_ (**F**) in the presence of 7.12 µM gentamicin. (**G**) Calculated values of *V*_in_ in XMP11 at *C*_0_ = MIC. The results are displayed as mean ± SEM of three biological replicates. * *P*＜0.05, ** *P*＜0.01, and *** *P*＜0.001.

One of the primary reasons for bacterial sensitivity lies in the ability of antimicrobial agents to accumulate at bactericidal concentrations within the bacterial cell (14). Therefore, efflux and influx kinetics of gentamicin were measured in the presence or absence of glutamate. Intracellular gentamicin concentration was elevated with increasing extracellular gentamicin doses in the synergy of gentamicin and glutamate, but only a slight elevation was observed in gentamicin alone (Fig. 3A). The increasing extracellular gentamicin also caused higher efflux in the synergy group than gentamicin alone (Fig. 3B). A similar trend was observed in the relationships of incubation period with intracellular drug concentration and efflux (Fig. 3C and D). Intracellular drug concentration increased in an extracellular glutamate dose-dependent manner (Fig. 3E), accompanied by enhanced efflux (Fig. 3F). We designated the relationship of drug efflux and drug influx as *V*_e_ = *V*_in_ when the extracellular concentration of gentamicin is equal to MIC so that the intracellular effective concentration of gentamicin is zero, as previously reported (15). *V*_e_ and *V*_in_ denote the velocity of efflux and influx, respectively. Under MIC concentration, glutamate complementation increased drug influx to 1.51 nmol/mg per second, which led to an approximately 7.38-fold increase in *V*_in_ (Fig. 3G). In addition, qRT-PCR showed that the expression of *ant(2'')-I*, *aac (3)-I*, *aac (3)-II*, and *aac(6')-I*, which encode aminoglycoside-modifying enzymes, was undetectable in this strain, despite the inclusion of a positive control.

### Analysis of glutamate flux by reprogramming metabolomics and isotope labeling

**Fig 4:**
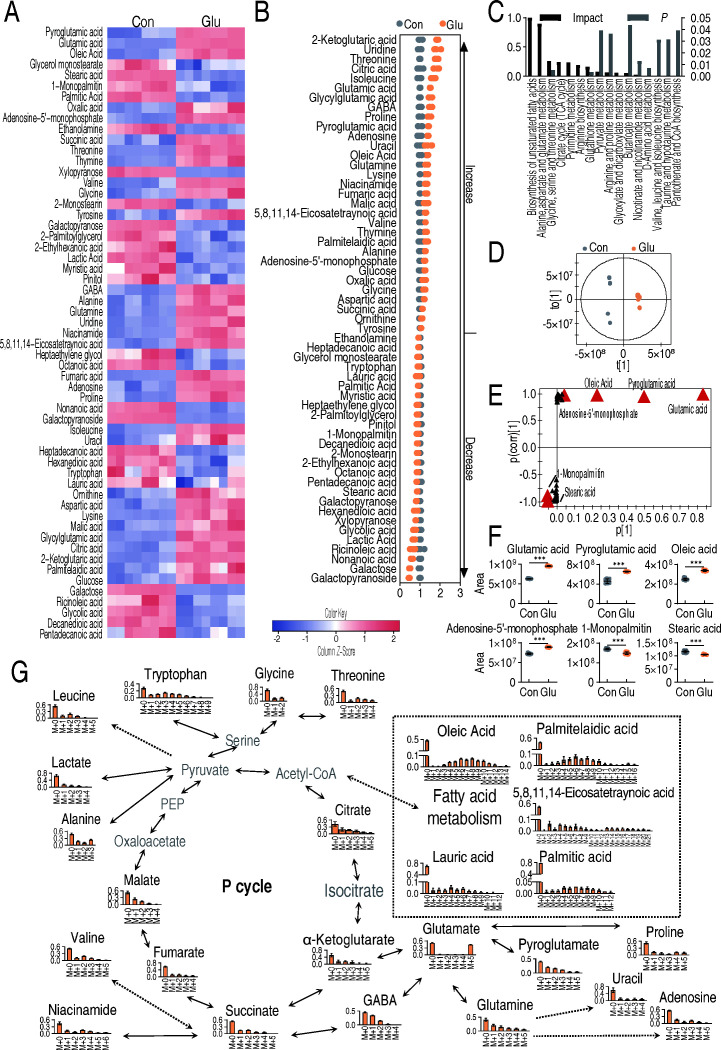
Analysis of glutamate flux through reprogramming metabolomics and isotope labeling. (**A**) The heatmap of the abundance of 57 differential metabolites between the control group and the glutamate group of XMP11. The color change from blue to red represents a change in metabolite abundance from low to high, and the heat map scale is shown at the bottom. (**B**) Fold change analysis of the 57 differential metabolites, corresponding to data in (**A**). (**C**) Enriched metabolic pathways (*P*＜0.05) of the 57 differential metabolites by the website analysis (https://www.metaboanalyst.ca/). (**D**) OPLS-DA of metabolomes between the control group and glutamate group of XMP11. Each dot represents the biological and technical replicates analysis of samples in the plot. (**E**) S-plot generated from OPLS-DA. Predictive component p[1] and correlation p(corr)[1] differentiate the control group from the glutamate group of XMP11. A triangle represents an individual metabolite, where potential biomarkers are highlighted with red, which is greater than or equal to 0.05 and 0.5 for the absolute value of covariance p and correlation p(corr), respectively. Otherwise, the triangle is marked in black. (**F**) The scatter plot of potential biomarkers in (**E**). The results are displayed as mean ± SEM of four biological replicates and two technical replicates. * *P*＜0.05, ** *P*＜0.01, and *** *P*＜0.001. (**G**) Mass isotopomer distributions for ^13^C_5_-labeled glutamate by non-target metabolomics.

To explore the metabolic fate of exogenous glutamate contributing to the glutamate-potentiated mechanisms, reprogramming metabolomics and isotope labeling were employed in the strain XMP11. By comparing metabolic profiles before and after glutamate treatments, the resultant changes in metabolite abundance were visualized using a heatmap (Fig. S3A). Fifty-seven metabolites were significantly altered following glutamate supplementation (Fig. 4A). The classification of these 57 metabolites was presented in Figure S3B, with 17% carbohydrates, 32% lipids, 28% amino acids, 9% nucleotides, and 14% others. Notably, lipids and amino acids together accounted for over 50% of the differential metabolites, underscoring their importance as the principal categories among these metabolites. Examination of the heatmap and fold change analysis revealed that for most metabolites, like amino acids and unsaturated fatty acids, abundance rose considerably following glutamate treatment (Fig. 4B).

Through metabolic pathway enrichment analysis, 16 enriched metabolic pathways were identified. Among them, biosynthesis of unsaturated fatty acids and alanine, aspartate, and glutamate metabolism exhibited the highest impact scores and emerged as the top two enriched metabolic pathways (Fig. 4C). A color scale diagram illustrating the abundance changes of these differential metabolites showed that the levels of saturated fatty acids (e.g., palmitic and stearic acids) decreased and those of unsaturated fatty acids increased within the biosynthesis of unsaturated fatty acids pathway; amino acid abundance rose in alanine, aspartate, and glutamate metabolism pathway (Fig. S3C). OPLS-DA revealed that t[1] effectively distinguished the control group from the glutamate-treated group (Fig. 4D). S-plot analysis showed that six biomarkers were identified, including glutamate, pyroglutamic acid, oleic acid, adenosine-5'-monophosphate (AMP), 1-monopalmitin, and stearic acid (Fig. 4E). Among them, the levels of glutamate, pyroglutamic acid, oleic acid, and AMP increased following glutamate treatment, whereas stearic acid and 1-monopalmitin exhibited decreases in abundance (Fig. 4F).

To further trace the metabolic fate of glutamate in XMP11, an isotopic-labeling experiment was conducted using an equimolar mixture (1:1) of unlabeled glutamate and ^13^C_5_-labeled glutamate, which enabled precise tracking of the labeled carbon atoms through various metabolic pathways. A total of 25 metabolites displaying isotopic peaks were identified, most of which belonged to the pyruvate cycle (16), amino acid metabolism, fatty acid biosynthesis, and, to a lesser extent, nucleotide metabolism. These findings collectively underscore the broad metabolic conversion of exogenous glutamate in XMP11, with clear evidence of glutamate-derived carbon contributing to central carbon metabolism, amino acid synthesis, and fatty acid production (Fig. 4G). Among the isotopically labeled fatty acids, oleic acid, palmitoleic acid, and arachidonic acid are unsaturated, whereas lauric acid and palmitic acid are saturated acids. The isotopic-labeling analysis revealed that exogenous glutamate was preferentially channeled into the biosynthesis of unsaturated fatty acids, with relatively minimal flux toward the production of saturated fatty acids. When these findings were combined with metabolomic analysis, showing increased levels of oleic, palmitoleic, and arachidonic acids, alongside decreased levels of lauric and palmitic acids following glutamate reprogramming, it becomes evident that one of the principal functions of glutamate reprogramming is to modulate fatty acid synthesis, specifically by elevating the abundance of unsaturated fatty acids and reducing that of saturated fatty acids.

### Impact of glutamate on fatty acid biosynthesis

**Fig 5:**
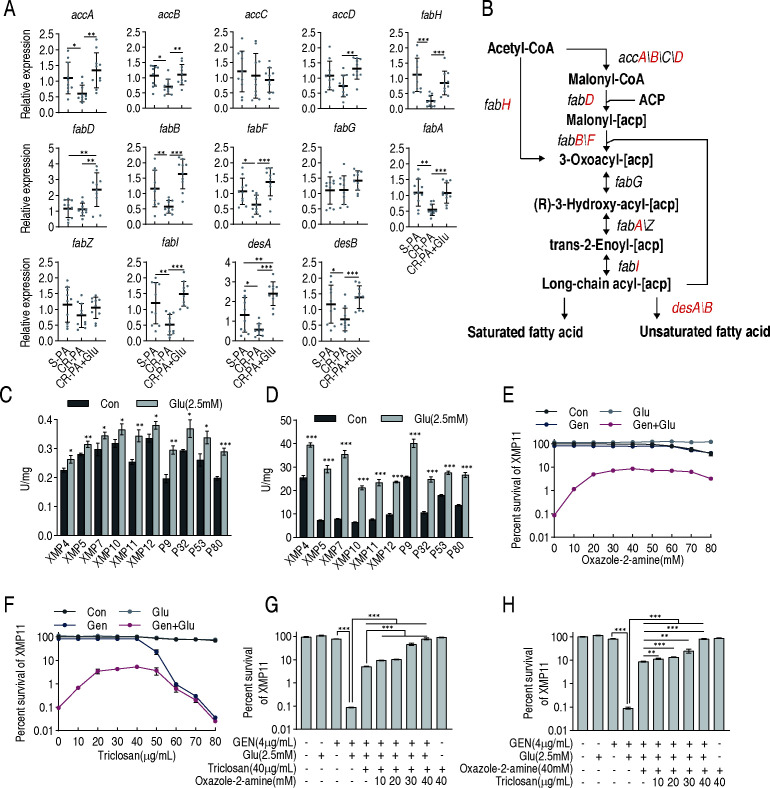
Impact of glutamate on fatty acid biosynthesis. (**A**) Expression levels of genes related to fatty acid biosynthesis among sensitive bacteria, carbapenem-resistant bacteria, and glutamate-treated carbapenem-resistant bacteria. (**B**) Schematic diagram of the distribution of each gene of (**A**). (**C**) Assay of GPT enzyme activity of 10 CR-PA without and with glutamate in M9 medium. (**D**) Assay of ACC enzyme activity of 10 CR-PA without and with glutamate in M9 medium. (**E**) The effect of oxazole-2-amine on the synergistic bactericidal action of glutamate and gentamicin in M9 medium. (**F**) The effect of triclosan on the synergistic bactericidal action of glutamate and gentamicin in M9 medium. (**G**) The effect of 40 µg/mL triclosan plus different concentrations of oxazole-2-amine on the synergistic bactericidal action of glutamate and gentamicin in M9 medium. (**H**) The effect of 40 mM oxazole-2-amine plus different concentrations of triclosan on the synergistic bactericidal action of glutamate and gentamicin in M9 medium. The results are displayed as mean ± SEM of three biological replicates. * *P*＜0.05, ** *P*＜0.01, and *** *P*＜0.001.

To elucidate the impact of glutamate on fatty acid biosynthesis, a multistep process governed by the coordinated regulation of numerous genes, qRT-PCR was employed to assess the expression of fatty acid biosynthesis genes in 10 antibiotic-sensitive *P. aeruginosa* (S-PA) and 10 CR-PA isolates with or without glutamate. The 10 S-PA strains include S1, S9, S14, S19, S24, S25, S26, S30, S32, and S34. The MIC data for these strains are shown in Table S2. Among the 14 genes tested without glutamate, nine (*accA*, *accB*, *fabH*, *fabB*, *fabF*, *fabA*, *fabI*, *desA,* and *desB*) were downregulated in CR-PA, and five (*accC*, *accD*, *fabD*, *fabG*, and *fabZ*) were not different. When glutamate was added to CR-PA, the expression of all 14 genes was elevated to be similar to or higher than S-PA (Fig. 5A and B). Note that *accA, accB, accC*, and *accD* together encode acetyl-CoA carboxylase (ACC). *desA* and *desB* serve for the biosynthesis of unsaturated fatty acids (17). We next measured the activities of two key enzymes that direct glutamate into the fatty acid biosynthesis pathway, namely glutamate-pyruvate transaminase (GPT) and ACC. GPT and ACC exhibited increased activity to varying degrees in the 10 CR-PA isolates following glutamate treatment, respectively (Fig. 5C and D).

Furthermore, two critical inhibitors targeting key enzymes in fatty acid biosynthesis, oxazole-2-amino and triclosan, which inhibit acetyl-CoA carboxylase (ACC) and enoyl-acyl carrier protein reductase (FabI), respectively, were used to validate the flux of glutamate into fatty acid biosynthesis. Inhibition rate was elevated with increasing oxazole-2-amino concentration before 20 mM and kept stable in 20−80 mM, increasing bacterial survival by up to 97.33-fold (Fig. 5E). Inhibition rate also increased in a triclosan dose-dependent manner during 0−20 mM, kept stable during 20−40 mM, and then descended in parallel with percent survival. The inhibition of glutamate-mediated potentiation increased bacterial survival by up to 56.82-fold at 40 mM (Fig. 5F). When 40 mM oxazole-2-amino was combined with a gradient of increasing triclosan concentrations or when 40 µg/mL triclosan was combined with increasing concentrations of oxazole-2-amino, bacterial survival increased and eventually reached a level comparable with the control when both inhibitors were present at 40 mM and 40 µg/mL, respectively (Fig. 5G and H). These observations are consistent with our isotopic-labeling studies, collectively indicating that exogenous glutamate upon entering central carbon metabolism ultimately flows into fatty acid biosynthesis.

### Glutamate-reprogramming lipidomics

**Fig 6:**
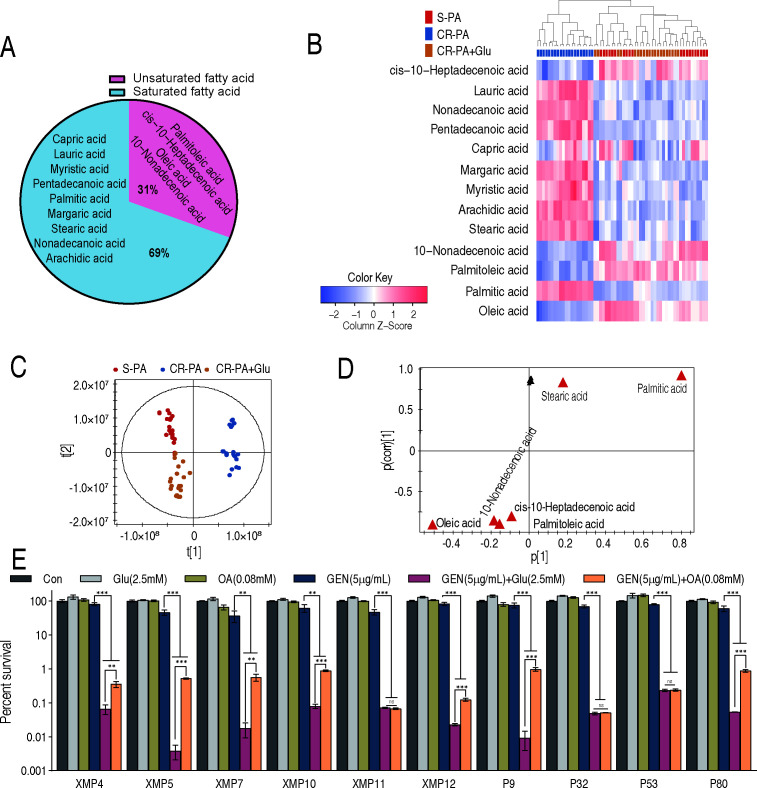
Glutamate-reprogramming lipidomics. (**A**) Classification of fatty acids identified in the fatty acid metabolome. (**B**) The heatmap of the abundance of 13 fatty acids among sensitive bacteria, carbapenem-resistant bacteria, and glutamate-treated carbapenem-resistant bacteria. The color change from blue to red represents a change in metabolite abundance from low to high, and the heat map scale is shown on the bottom. (**C**) OPLS-DA of metabolomes among sensitive bacteria, carbapenem-resistant bacteria, and glutamate-treated carbapenem-resistant bacteria. Each dot represents the biological and technical replicates analysis of samples in the plot. (**D**) S-plot generated from OPLS-DA. Predictive component p[1] and correlation p(corr)[1] differentiate sensitive bacteria, carbapenem-resistant bacteria, and glutamate-treated carbapenem-resistant bacteria. A triangle represents individual metabolite, where potential biomarkers are highlighted in red, which is greater than or equal to 0.05 and 0.5 for the absolute value of covariance p and correlation p(corr), respectively. Otherwise, the triangle is marked in black. (**E**) Comparison of the bactericidal effects of glutamate and oleic acid on promoting gentamicin in M9 medium. The results are displayed as mean ± SEM of three biological replicates. * *P*＜0.05, ** *P*＜0.01, and *** *P*＜0.001.

The above results indicate that exogenous glutamate fluxes to fatty acid biosynthesis, possibly promoting the biosynthesis of unsaturated fatty acids. To clarify this, GC-MS-based lipidomics was carried out on three groups: 10 S-PA, 10 CR-PA without (CR-PA), and 10 CR-PA with glutamate (CR-PA + Glu). After removing the internal standard (methyl tridecanoate) and excluding artificial peaks, a total of 13 fatty acids were identified, comprising 9 saturated and 4 unsaturated fatty acids (approximately 69% and 31%, respectively) (Fig. 6A). A heatmap was generated to visualize fatty acid abundances in each group. Interestingly, S-PA and CR-PA + Glu were clustered together and were clearly separated from CR-PA. Among the 9 saturated and 4 unsaturated fatty acids, saturated fatty acids (all except capric acid) were more abundant and unsaturated fatty acids were less abundant in CR-PA compared with CR-PA + Glu and S-PA (Fig. 6B). OPLS-DA revealed that t[1] effectively distinguished CR-PA from S-PA and CR-PA + Glu (Fig. 6C). S-plot identified six biomarkers, including four unsaturated fatty acids and two saturated fatty acids. Among the four unsaturated fatty acids, oleic acid was identified as the most significant biomarker, as it exhibited the lowest values of both p and p(corr) in the S-plot analysis (Fig. 6D). These results suggest that oleic acid is the key biomarker in the lipidome impacted by glutamate metabolic flux. To support this conclusion, the survival of 10 CR-PA isolates was measured in the presence of gentamicin with oleic acid and with glutamate as a positive control. Compared with gentamicin alone, low viability was detected in all isolates in the synergistic use of gentamicin with oleic acid or glutamate. Particularly, comparable viability was observed in three isolates (XMP11, P32, and P53), whereas higher survival was detected under the synergy with oleic acid than with glutamate in the remaining seven isolates (Fig. 6E).

### Mechanisms by which glutamate promotes intracellular drug concentration

**Fig 7:**
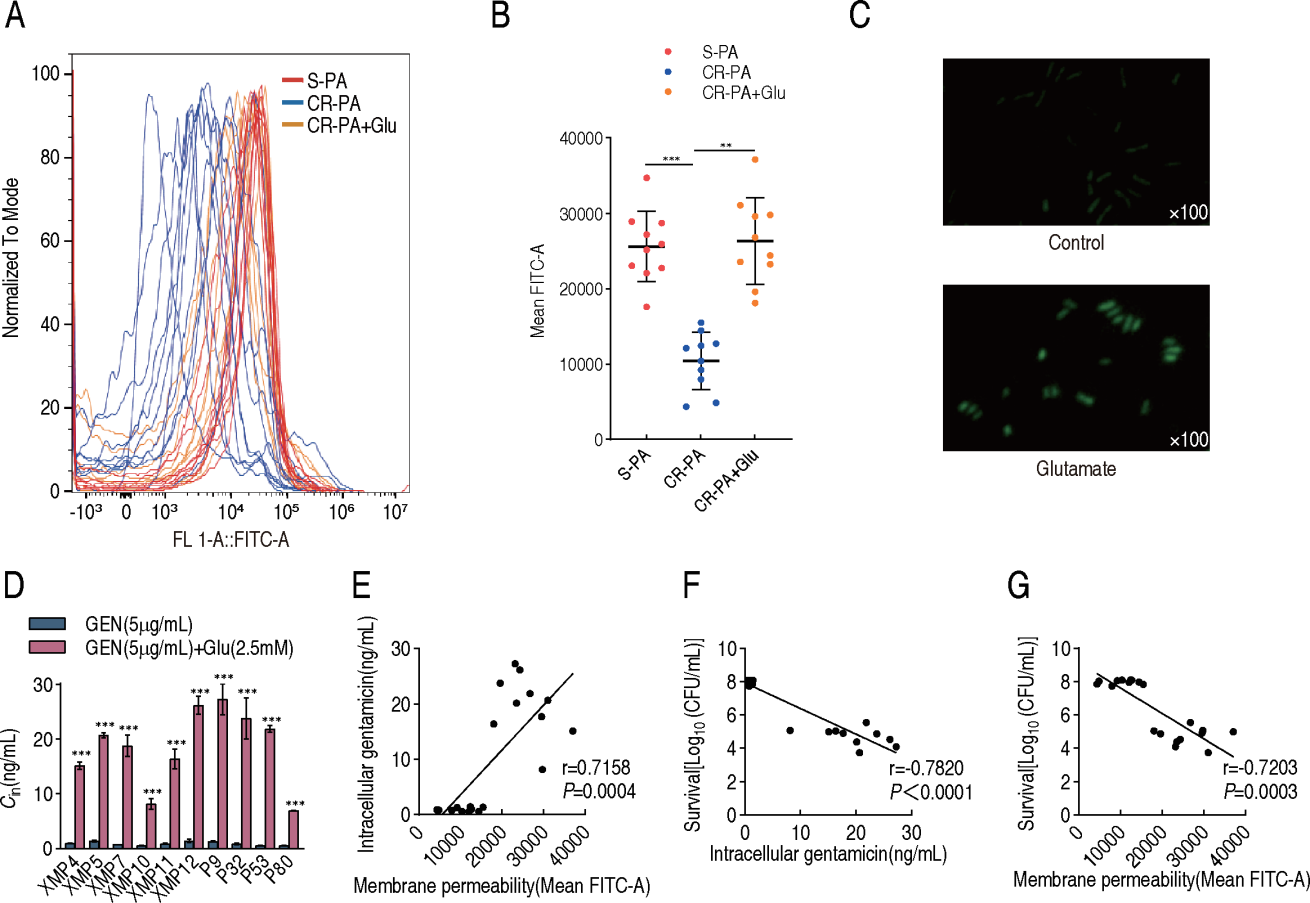
Mechanisms by which glutamate promotes intracellular drug concentration. (**A**) Determination of membrane permeability of sensitive bacteria, carbapenem-resistant bacteria, and glutamate-treated carbapenem-resistant bacteria. (**B**) Mean FITC-A value of membrane permeability. (**C**) Membrane permeability of XMP11 in the absence or presence of 2.5 mM glutamate in M9 medium, detected in fluorescence microscopy. (**D**) Intracellular gentamicin concentration with or without glutamate in the 10 CR-PA. (**E**) Analysis of the correlation between membrane permeability and intracellular gentamicin. (**F**) Analysis of the correlation between intracellular gentamicin and bacterial survival. (**G**) Analysis of the correlation between membrane permeability and bacterial survival. The results are displayed as mean ± SEM of three biological replicates. * *P*＜0.05, ** *P*＜0.01, and *** *P*＜0.001.

Reports have shown that the ratio of unsaturated to saturated fatty acids is related to membrane permeability (14). We therefore investigated whether the glutamate-induced changes in fatty acid composition translate into increased membrane permeability and gentamicin uptake. Membrane permeability was reduced in CR-PA compared with S-PA, but this reduction was reversed by exogenous glutamate (Fig. 7A and B). The reversed membrane permeability was further demonstrated by fluorescence microscopy (Fig. 7C). Consistently, exogenous glutamate increased the intracellular gentamicin concentration in all 10 CR-PA strains (Fig. 7D). Correlation analysis demonstrated that intracellular gentamicin was positively correlated with membrane permeability (r = 0.7158) (Fig. 7E), whereas bacterial survival was negatively correlated with both intracellular gentamicin (r = −0.7820) (Fig. 7F) and membrane permeability (r = −0.7203) (Fig. 7G).

## DISCUSSION

The predicament of eradicating MDR-PA lies in its low permeability of the outer membrane (18), which is exacerbated by carbapenem resistance (19). This is why increasing the membrane permeability can become an effective strategy for developing next-generation antibiotics (14, 20). Thus, it is especially crucial to increase intracellular antibiotic concentration through promoting drug uptake for combating this pathogen. However, restoring or increasing the permeability of the cell membrane in antibiotic-resistant bacteria remains a major challenge. Advances in reprogramming metabolic states have made it possible to increase drug uptake via reprogramming metabolites (6, 21–23). Thus, the present study employs this strategy to identify reprogramming metabolites that reverse the antibiotic-resistant metabolic state into an antibiotic-sensitive one for increasing gentamicin uptake. Glutamate was identified as the ideal reprogramming metabolite to promote gentamicin-mediated killing of lab-evolved gentamicin-resistant PA and clinical MDR-PA and CR-PA isolates. The underlying mechanisms involve promoting the biosynthesis of unsaturated fatty acids and inhibiting the biosynthesis of saturated fatty acids through glutamate metabolism. These findings not only provide an effective reprogramming metabolite against MDR-PA and CR-PA but also reveal a previously unknown glutamate-biosynthesis of unsaturated fatty acid-membrane permeability metabolic regulatory pathway that is involved in gentamicin sensitivity.

Metabolic reprogramming approaches have been utilized to combat antibiotic-resistant *P. aeruginosa* (11, 24–26), but until now, the reprogramming metabolite-enabled antibiotic potentiation has not been applied to clinical *P. aeruginosa* isolates. Here, suppressed glutamate in antibiotic-resistant *P. aeruginosa* was identified as a reprogramming metabolite. Interestingly, a lower level of glutamate was identified as one of the five biomarkers distinguishing drug-resistant from drug-sensitive *P. aeruginosa* isolated from cystic fibrosis patients (27), supporting glutamate as a metabolic state-reprogramming molecule to reprogram the multidrug-resistant state into an antibiotic-sensitive state. Both *in vitro* and *in vivo* experiments showed that exogenous glutamate boosts gentamicin-mediated killing of not only clinically isolated MDR-PA but also CR-PA. CR-PA has been listed by the WHO as one of the most threatening pathogens for which novel antibiotics are urgently needed. Therefore, the present study proposes a previously unrecognized therapeutic strategy to combat CR-PA.

In clinical settings, insufficient antibiotic concentrations at the site of infection that kill or suppress bacterial growth are a major cause of antibiotic treatment failure (28), which, in turn, results in inadequate intracellular antibiotic concentrations within bacteria. Common types of infections caused by *P. aeruginosa* include respiratory tract infections, bloodstream infections, urinary tract infections, and wound infections (2). Hydrogels have become the most competitive candidates for wound dressings due to their good hydrophilicity, biocompatibility, and three-dimensional (3D) porous structure like extracellular matrix (ECM) (29). There have been reports on hydrogels containing aminoglycoside antibiotics (30, 31). It may be possible to develop a hydrogel capable of simultaneously releasing glutamate and gentamicin, thereby achieving high local concentrations of both glutamate and gentamicin at wound sites. Liposomes and PLGA (poly lactic-co-glycolic acid) nanospheres are commonly used to construct drug delivery systems (32, 33). Gentamicin, high-concentration glutamate, and nanospheres can be co-formulated into a drug delivery system. On one hand, this approach prevents the degradation of gentamicin *in vivo* (34). On the other hand, the encapsulated high-concentration glutamate could be released simultaneously with gentamicin, creating a local microenvironment with elevated glutamate levels around the bacteria.

The intracellular drug concentration is dependent on the balance between uptake and dampening. Low membrane permeability limits the entry of extracellular antibacterial agents, whereas efflux and enzymatic degradation contribute to the consumption of intracellular antibiotics (14). Since the intracellular concentration of antibiotics is approximately 20% lower in antibiotic-resistant bacteria than in antibiotic-sensitive bacteria (21, 35), the reduction of bacterial survival by more than 100-fold following exogenous glutamate complementation cannot result from the consumption of intracellular drug. Instead, it should be attributed to the elevated uptake. Namely, exogenous glutamate stimulates drug uptake to an extent that overcomes efflux. Ultimately, this could increase the intracellular drug level to a lethal concentration that causes more than a 100-fold killing. This concept is demonstrated through measurements of influx and efflux in the present study. Therefore, the elevation of membrane permeability by metabolic reprogramming is an effective solution against CR-PA.

Aminoglycosides are still considered a critically important class of antibacterial agents (36). The uptake of aminoglycoside antibiotics is believed to involve the penetration through the outer membrane and the energy-dependent entry into the inner membrane (37). Reduced outer membrane permeability is typically associated with decreased intracellular antibiotic concentrations (38, 39), which is often one of the most common mechanisms of aminoglycoside resistance (21). However, why membrane permeability is reduced and how to reverse it are largely unknown. The present study shows that the reduction of membrane permeability is attributed to the suppression of unsaturated fatty acids and the elevation of saturated fatty acids. Interestingly, exogenous glutamate metabolic flux can increase the biosynthesis of unsaturated fatty acids and decrease the biosynthesis of saturated fatty acids. This change elevates the membrane permeability in MDR-PA and CR-PA, increasing intracellular drug concentration and thereby significantly enhancing bactericidal efficiency. These findings are consistent with previous reports on the relationship between saturated/unsaturated fatty acids and membrane permeability (40–43). In most bacteria, including *P. aeruginosa*, fatty acids have essentially no alternative metabolic fates since nearly all long-chain fatty acids produced by biosynthetic pathways are directly incorporated into cellular membranes (40). The structural characteristics of these fatty acids determine key membrane properties (41): straight-chain saturated fatty acids (e.g., stearic acid) adopt linear conformations that enable tight packing, forming bilayers with high phase transition temperatures and low permeability, whereas unsaturated fatty acids introduce distinct kinks in the acyl chains that disrupt dense packing, resulting in more disordered membrane arrangements with increased permeability (40). Bacteria can modulate membrane unsaturation through two primary mechanisms: direct incorporation of exogenous unsaturated fatty acids into the lipid bilayer or enzymatic desaturation of existing saturated chains (42). Notably, exposure to exogenous polyunsaturated fatty acids induces significant phospholipid remodeling in *P. aeruginosa*, with these fatty acids being preferentially incorporated into the major membrane phospholipids phosphatidylethanolamine and phosphatidylglycerol, leading to increased membrane permeability (43). Therefore, fatty acid biosynthesis plays a critical role in maintaining membrane homeostasis as the biophysical properties of membranes are largely determined by the composition of their constituent fatty acids.

Interestingly, the effective glutamate concentration in our *in vitro* experiments (2.5 mM) far exceeds physiological plasma levels in humans (0.05−0.1 mM). This discrepancy is common in metabolic reprogramming studies. For instance, glutamine synergizes with ampicillin against multidrug-resistant uropathogenic bacteria at *in vitro* concentrations (20 mM) vastly higher than human physiological levels (0.371−0.957 mM) (6, 44). However, *in vivo* efficacy often requires lower doses: although glutamine potentiates ampicillin at 100 mg/kg in mice, our study used a slightly higher glutamate dose (200 mg/kg). These *in vitro* vs. *in vivo* disparities highlight the complexity of metabolic regulation *in vivo*, warranting further investigation.

In summary, the metabolic reprogramming approach is used to explore the combination of a reprogramming metabolite and gentamicin against MDR-PA and CR-PA. Glutamate potentiates gentamicin killing against MDR-PA and CR-PA *in vitro* and *in vivo*. This is because exogenous glutamate increases the biosynthesis of unsaturated fatty acids and reduces the biosynthesis of saturated fatty acids to elevate membrane permeability for promoting gentamicin uptake. Therefore, this study provides a drug candidate to eliminate MDR-PA and CR-PA.

## MATERIALS AND METHODS

### Bacterial strains and culture conditions

ATCC 27853 and clinical strains of *P. aeruginosa* were obtained from our lab stock. Separated by streak plate method on Luria-Bertani (LB) agar plate, three colonies were selected to culture at 37°C and shake at 200 rpm for 16 h in LB broth. To select for gentamicin resistance, a PA-R_GEN_ strain was sequentially generated from ATCC 27853 in the test tubes with 5 mL LB broth plus 0.5 MIC of gentamicin (1  µg/mL) until its MIC reached up to 8 MIC (16  µg/mL). A control gentamicin-sensitive *P. aeruginosa* strain (PA-S) was generated in the same way except for gentamicin. The sequential cycles resulted in the development of PA-R_GEN_ and PA-S.

### MIC assay

The minimum inhibitory concentrations (MICs) were assessed using the CLSI broth microdilution method. The stationary-phase samples were re-inoculated at a 1:100 dilution into Mueller-Hinton (MH) broth to a 0.5 McFarland turbidity standard. The bacterial samples were further diluted with fresh MH broth to reach a cell density of 5 × 10^6^ CFU/mL. Then, 10 µL of the bacterial suspension was added to 100 µL of MH broth containing a gradient of antibiotic concentrations in the 96-well plate, bringing the final inoculum to 5 × 10^5^ CFU/mL. The 96-well plate was then statically incubated at 37°C for 16 h, and the lowest concentration, which inhibited the visual growth, was recorded as MIC.

### Growth curve measurement

The stationary-phase cultures were re-inoculated at a 1:100 dilution into fresh LB broth at 37°C and 200  rpm. The values of OD_600_ were measured at 0, 2, 4, 6, 8, 10, and 12  h. The growth curve under these conditions was generated by plotting the values of OD_600_ and the corresponding culture time.

### GC–MS analysis

GC–MS analysis was conducted as previously described (6, 41). Briefly, overnight cultures were washed three times with physiological saline, resuspended in M9 medium containing the corresponding treatment, and incubated at 37°C with shaking at 200  rpm for 6 h. The cultures were then centrifuged at 8,000  rpm for 5 min, washed three times with physiological saline, and resuspended to an OD600 of 1.0. A 10  mL aliquot of these cultures was collected and resuspended in 1  mL of pre-cooled methanol (−80°C, Sigma). For normalization, an internal standard (0.1  mg/mL ribitol, Sigma) was added. The cells were disrupted by sonication (35% power, 2  s on / 3  s off) for 10 min. After centrifugation at 12,000  rpm and 4°C for 10 min, 900  µL of the supernatant was transferred to a new tube and dried completely in a vacuum concentrator at 37°C (approximately 180 min). Subsequently, 80  µL of methoxylamine hydrochloride (20  mg/mL in pyridine) was added, followed by ultrasonication to dissolve the precipitate and incubation at 37°C for 180 min. Then, 80  µL of N-methyl-N-(trimethylsilyl)trifluoroacetamide (MSTFA) was added, and the mixture was incubated at 37°C for 30 min. After centrifugation at 12,000  rpm for 10 min, 120  µL of the supernatant was transferred to an insert vial for injection. A 1  µL aliquot of the derivatized sample was injected into a DB-5 MS column. The initial oven temperature was held at 85°C for 5 min, then increased to 270°C at a rate of 15°C/min, and held for 5 min. Helium was used as the carrier gas at a constant flow rate of 1  mL/min. Mass spectrometry data were acquired in the range of 50−600  m/z. The analysis was performed using an Agilent 7890A GC system coupled with an Agilent 5975C VL MSD detector (Agilent Technologies).

### Analysis of metabolomic data

Analysis of metabolomics data was carried out as previously described (45). Initial peak detection and mass spectral deconvolution were carried out with Agilent software (Agilent 6.0). Metabolites were identified according to spectral matching and retention time (RT) by searching in the National Institute of Standards and Technology (NIST) Mass Spectral Library. The data matrix was normalized using an internal standard (ribitol) and the total intensity. Software IBM SPSS Statistics 26 was used to analyze statistical differences, and a value of *P*  <  0.01 was considered significant. Hierarchical clustering was completed in the R platform with the package gplots1 using the distance matrix. Fold-change analysis was used to analyze the normalized area of differential metabolites. Multivariate statistical analysis included Orthogonal Partial Least Squares Discriminant Analysis (OPLS-DA), and S-plot analysis was performed by SIMCA-P  + 12.0.1 software (Umetrics, Umea, Sweden). Enrichment of significant metabolic pathways was conducted with MetaboAnalyst 6.0 (13). GraphPad Prism 8.0 was used to draw figures.

### Antibiotic bactericidal assay

Antibacterial assay was carried out as previously described with a modification (24). Three single colonies were selected to culture at 37°C and 200 rpm for 16 h in LB broth to prepare the stationary-phase cultures. The bacterial cells were harvested by centrifugation and suspended in M9 medium to an OD_600_ of 0.200 ± 0.005. Desired concentrations of antibiotics and glutamate were added and then incubated at 37°C, 200  rpm for 6 h. To determine bacterial counts, 100  µL of the cultures was acquired and serially diluted. Aliquot of 5  µL of each dilution was spotted on LB agar plates and cultured at 37°C for 18 h. The plates only yielding 20−200 colonies were counted, and CFU/mL was calculated. Biological replicates were performed in triplicate.

### Biofilms

We followed the methods described by Zhao et al. for the cultivation of biofilms (6). The stationary-phase samples of PA-R_GEN_ were re-inoculated at 1:100 dilutions into 3 mL LB broth, which includes PE50 tubes (0.58 mm × 0.96 mm). The tubes were left static in an incubator set at 37℃, and the fresh LB broth was replaced at a 1:1 ratio every 24 h for 3 consecutive days. The PE50 tubes were washed five times with saline to remove planktonic bacteria. Then, eight tubes were transferred to a 1.5 mL EP tube, with the addition of metabolites and antibiotics. M9 medium was added to achieve a final reaction system volume of 500 µL. The reaction mixture was then allowed to incubate on a shaker incubator at 37°C for 6 h. After incubation, the tubes were washed with saline to remove planktonic bacteria and transferred into a new EP tube with 1 mL saline. The bacterial suspension was then subjected to water bath ultrasonic treatment for 10 min, followed by vortexing for 1 min, and finally, a serial dilution was used to obtain bacterial counts.

### Quantitative real-time PCR

To investigate the effect of glutamate on gene expression levels, quantitative real-time PCR (qRT-PCR) was performed as previously described (46). In brief, after incubation in M9 with or without glutamate at 37°C with 200 rpm for 6 h, the cells were collected and adjusted to an OD_600_ of 1.0. Total RNA was isolated from 1 mL of cell samples by TRIzol reagent (Ambion). cDNA was obtained from 1 µg total RNA, and reverse transcription was performed according to a PrimeScript RT reagent kit with gDNA Eraser (Guangzhou IGE Biotechnology Ltd., China). The primers used for qRT-PCR are listed in Table S3, with the 16S rRNA gene serving as an internal control. The target genes include those associated with the fatty acid biosynthesis pathway, as well as aminoglycoside-modifying enzyme genes *ant(2'')-I, aac (3)-I, aac (3)-II*, and *aac(6')-I*. The qRT-PCR was performed in 384-well plates with the SYBR Green Premix Pro Taq HS qPCR Kit (Guangzhou IGE Biotechnology Ltd., China) at a total volume of 10  µL. The reaction mixtures were run on a LightCycler 480 system (Roche, Germany). The cycling parameter values were set as follows: 95°C for 30 s to activate the polymerase, 40 cycles of 95°C for 5 s, 58°C for 30 s. Fluorescence measurements were performed at 75°C for 1 s during each cycle. Cycling was terminated at 95°C with a calefactive velocity of 0.11°C/s to obtain a melting curve. Data were calculated as relative mRNA expression compared to without nitrite group with the endogenous reference 16S rRNA gene.

### Mouse experiment *in vivo*

A mouse experiment *in vivo* was carried out as described previously (6). Three single colonies were inoculated into 50 mL of LB medium and cultured overnight. The bacterial suspension was aliquoted into several EP tubes containing an appropriate amount of glycerol. BALB/c mice were used in this experiment. For mouse experiments, a fixed volume of 50 µL glycerol-preserved bacterial suspension was added to 50 mL LB medium and cultured at 37°C, 200 rpm for 16 h. The culture was then washed three times with saline and resuspended in saline to adjust the OD_600_ to 0.400. A total of 100 µL of the bacterial suspension was intraperitoneally injected into each mouse, with an approximate bacterial dose of 1.47 × 10^7^ CFU per mouse. Treatment was administered an hour post-injection, with mice divided into four groups: the saline group, the glutamate group, the gentamicin group, and the gentamicin plus glutamate group. The treatment dose was 200 mg/kg for glutamate and 10 mg/kg for gentamicin. Survival was monitored for 48 h. For bacterial load quantification in mouse organs, the liver, kidney, and spleen samples were collected 6 h after treatment. The organs were homogenized using a tissue grinder with an appropriate amount of saline, followed by serial dilution in saline and plate counting to determine bacterial colony counts.

### Measurement of intracellular gentamicin

Three single colonies were selected to culture at 37°C and 200 rpm for 16 h in LB broth. Bacterial cultures were centrifuged at 8,000 rpm for 3 min at 24°C. The cells were washed three times with physiological saline, each time centrifuging at 8,000 rpm for 3 min at 24°C, and resuspended in M9 medium to an OD_600_ of 0.200 ± 0.005. Desired concentrations of gentamicin and glutamate were added to a test tube containing 5 mL bacterial suspension. After incubation at 37°C, 200 rpm for 6 h, the bacterial cells were collected by centrifugation and washed three times with physiological saline; 1 mL of bacterial culture resuspended with physiological saline at OD_600_ = 0.200 was collected. Crushed with an ultrasonic disruptor for 3 min, power at 35%, on for 2 s, off for 3 s. Centrifuged at 12,000 rpm at 4°C for 10 min. In total, 900 µL of the supernatant was transferred to a new centrifuge tube and diluted to the appropriate ratio. A gentamicin detection kit (Shanghai Jianglai Biotechnology, Shanghai, China, JL17568) was used to determine the gentamicin concentration according to the kit instructions. The final gentamicin concentration measured using the assay kit, multiplied by the dilution factor, represents the intracellular gentamicin concentration.

### Influx and efflux kinetics

The kinetics was carried out as described previously (6, 15). The efflux kinetics of XMP11 were determined at different extracellular antibiotic concentrations. The efflux rate *V*_e_ was calculated using the Michaelis-Menten equation {*V*_e_ = *V*_emax_ × (*C*_p_)^h^/[(*K*_0.5_)^h^ + (*C*_p_)^h^]}, where *V*_emax_ is the maximal efflux rate of 0.37 nmol·mg^−1^·s^−1^. *K*_0.5_ is the half-maximal flow rate drug concentration of 0.94 µM, and h is the Hill coefficient of 0.23. *C*_p_ was calculated as the intracellular gentamicin content determined above. In the absence of exogenous glutamate, *V*_in_ was calculated using the formula *V*_in_ = P × A× (*C*_0_−*C*_p_). The *C*_0_ value is the concentration of gentamicin in the XMP11 at 1 MIC value. When glutamate is added, *V*_in_ can be calculated as *V*_in_ = P × A × (*C*_p_ with glutamate/*C*_p_ without glutamate) × (*C*_0_ − *C*_p_). In the above equation, P is the osmotic coefficient (0.28 × 10^−5^ cm/s), and A is the cell surface area (constant value, 103 cm^2^/mg).

### Isotope-tracing analysis

Isotope-tracing analysis was carried out as described previously (47, 48). To explore the metabolic flow of glutamate in *P. aeruginosa*, we established two experimental groups in the strain XMP11. The first group was treated with 2.5  mM unlabeled glutamate, whereas the second group was treated with 2.5 mM glutamate consisting of an equal mixture of unlabeled and ^13^C_5_-labeled glutamate. The bacterial suspensions were washed with saline and adjusted with M9 to an OD₆₀₀ of 0.200 and subjected to the respective treatments. The cultures were then incubated at 37°C with shaking at 200 rpm for 6 h. The incubated bacteria were washed and adjusted to an OD_600_ of 1.000. Subsequently, 10 mL of bacterial suspension was collected by centrifugation at 8,000 rpm and immediately treated with 1 mL of cold methanol (HPLC grade, Thermo Fisher Scientific Company) to stop bacterial metabolic activity. As an internal standard, 10 µL of ribitol solution (0.1 mg/mL) was added to the cell suspension, which was sonicated for 20 min at 200 W operating power. The sonicated fractions were centrifuged at 4°C, 12,000 rpm for 10 min. The supernatant was transferred to a vacuum centrifuge dryer (Labconco, USA) at 37°C, and methanol was removed by evaporation. The dried sample was added with 80 µL of 20 mg/mL methoxime pyridine hydrochloride (Sigma-Aldrich), dissolved by ultrasonication, and reacted at 37°C for 3 h. Subsequently, 80 µL of N-methyl-N-trimethylsilyltri fluoroacetamide (MSTFA, Sigma-Aldrich) was added, which was carried out at 37°C for 45 min. GC-MS data were obtained using an Agilent 7890A gas chromatograph equipped with an Agilent 5975C VL MSD detector (Agilent Technologies). Aliquot with 1 µL of sample was injected into a 30 m × 250 mm (inner diameter), 0.25 mm DBS-MS column. The gas chromatography was held at an initial temperature of 85°C for 5 min, then ramped up to 270°C at a rate of 15°C/min, and held for 5 min. Helium was used as the carrier gas at a flow rate of 1 mL/min. The MS was operated in the range of 50−600 m/z. Four biological and two technical repeat sequences were prepared for each sample. Ion fragmentation spectra were analyzed, using the 2008 version of the NIST mass spectral library. Graphs were created using GraphPad Prism 8.0.

### Assay of enzyme activity

The experiment was performed using glutamic-pyruvic transaminase (GPT) and acetyl-CoA carboxylase (ACC) activity assay kits (Glutamic-pyruvic Transaminase [GPT] Activity Assay Kit, Solarbio, BC1555; Acetyl CoA carboxylase [ACC] Activity Assay Kit, Solarbio, BC6025). Ten strains of CR-PA were treated with or without 2.5 mM glutamate. The bacterial suspension was incubated at 37°C, 200 rpm for 6 h. They were washed three times and adjusted to OD_600_ = 1.000, respectively.

In total, 30 mL of the above bacterial culture of each strain was collected and resuspended in 1 mL of the corresponding sample dissolution buffer from the assay kit. The suspension was subjected to ultrasonic disruption for 20 min at 35% power, with a cycle of 2 s on and 3 s off. The lysate was then centrifuged at 12,000 rpm for 10 min at 4°C, and the supernatant was collected for subsequent enzymatic activity measurements according to the kit instructions.

### GC-MS-based lipidomics

Sample preparation was carried out as previously described (11). In brief, after being washed with physiological saline three times, the overnight cultures were resuspended in M9 medium with or without 2.5 mM glutamate and cultured at 37°C, 200 rpm for 6 h. Then, the cultures were centrifuged at 8,000 rpm for 5 min, washed three times, and resuspended with physiological saline and adjusted OD_600_ to 1.0. 10 mL of the collected bacterial cells were centrifuged at 12,000 rpm for 10 min, and the supernatant was discarded. The pellets were immediately quenched with liquid nitrogen and then resuspended in 1 mL of ultrapure water. A total volume of 10 mL of a methanol and methyl tert-butyl ether mixture (1:1, vol/vol) was added, followed by 20 µL of methyl tridecanoate as the analytical internal standard. The resulting suspension was subjected to ultrasonic disruption for 10 min at 35% power, employing a 2 s on, 3 s off cycle. The lysate was centrifuged at 12,000 rpm for 5 min at 4°C, and the supernatant was collected and evaporated to dryness in a rotary vacuum centrifuge. The residue was dissolved in 200 µL of hexane, after which 200 µL of a methanol solution containing 1 M KOH was added. The solution was hydrolyzed at 60°C for 30 min, and then 200 µL of a methanol solution containing 14% boron trifluoride was introduced. The sample was derivatized at 60°C for an additional 30 min, dried under vacuum, and redissolved in 1 mL of hexane. Next, 200 µL of saturated NaCl solution was added, along with anhydrous Na₂SO₄ to remove any residual moisture. An 800 µL aliquot of the upper layer was transferred to a fresh tube, dried again under vacuum, and finally reconstituted in 100 µL of hexane, which was placed in an injection vial. For gas chromatography (GC), the oven temperature was initially maintained at 85°C for 3 min, then programmed to rise at 10°C per minute to 285°C, where it was held for 10 min. The injection volume was 1 µL (splitless), and the total run time was 31.25 min. The solvent delay was 5 min, and high-purity helium (99.99%) served as the carrier gas at a flow rate of 30 mL/min. Mass spectrometry (MS) conditions were as follows: an ionization voltage of 70 eV, an acquisition mass range of m/z 50−560, and a scan time of 0.32 s.

### Assay of membrane permeability

As previously described, the permeability of bacterial cell membranes was measured (14). In brief, the bacteria were resuspended with M9 medium and adjusted to 0.200 of OD_600_; 2.5 mM glutamate was applied according to the designated group assignments. Then, the bacterial cells were incubated at 37°C, 200 rpm for 6 h. An aliquot of 100 µL of the sample was mixed with 900 µL of M9 medium. Next, 1 mL of this suspension was mixed with 2 µL of SYTO-9 green fluorescent dye (Invitrogen, USA) to achieve a final dye concentration of 50 µM, followed by incubation at 37°C in the dark for 15 min. The samples were then immediately transferred to flow cytometry tubes, and green fluorescence intensity was measured by flow cytometry. For higher fluorescence intensity, indicating greater membrane permeability, these values provided a quantitative measure of the cells’ membrane integrity.

### Measurement of permeability by fluorescence microscopy

As previously described, the permeability of bacterial cell membranes was measured by fluorescence microscopy (14). In brief, the bacteria, XMP11, were resuspended with M9 medium and adjusted to 0.200 of OD_600_; 2.5 mM glutamate was applied according to the designated group assignments. Then, the bacterial cells were incubated at 37°C, 200 rpm for 6 h. An aliquot of 100 µL of the sample was mixed with 900 µL of the M9 medium. Next, 1 mL of this suspension was mixed with 2 µL of SYTO-9 green fluorescent dye (Invitrogen, USA) to achieve a final dye concentration of 50 µM, followed by incubation at 37°C in the dark for 15 min. Then, the samples were centrifuged for 10 min at 12,000 rpm. The pellets were resuspended with 20 µL of PBS. An aliquot of the 2 µL sample was dropped on the agarose slide, and photos were taken under the inverted fluorescence microscope.

### Analysis of correlation

SPSS 26.0 was used to conduct the analysis of correlation. Normal analysis was performed for each set of data. Data on intracellular gentamicin and bacterial survival did not match the condition of normal analysis. Accordingly, the following analysis was based on the Spearman correlation.

## Data Availability

The metabolomics data have been deposited to MetaboLights repository with the study identifier MTBLS12656 (49).

## SUPPLEMENTAL MATERIAL

Supplemental material msystems.01234-25-s0001.docx Supplemental figures and tables.

ASM does not own the copyrights to Supplemental Material that may be linked to, or accessed through, an article. The authors have granted ASM a non-exclusive, world-wide license to publish the Supplemental Material files. Please contact the corresponding author directly for reuse.
