# Supplementary material for: Exogenous glutamate potentiates gentamicin to kill multidrug- and carbapenem-resistant Pseudomonas aeruginosa by activating the biosynthesis of unsaturated fatty acids
Source: mSystems. 2025 Oct 13;10(11):e01234-25. doi: 10.1128/msystems.01234-25 (PMC12625761; doi:10.1128/msystems.01234-25)
Supplement: Supplemental material — Supplemental figures and tables. [file msystems.01234-25-s0001.docx]

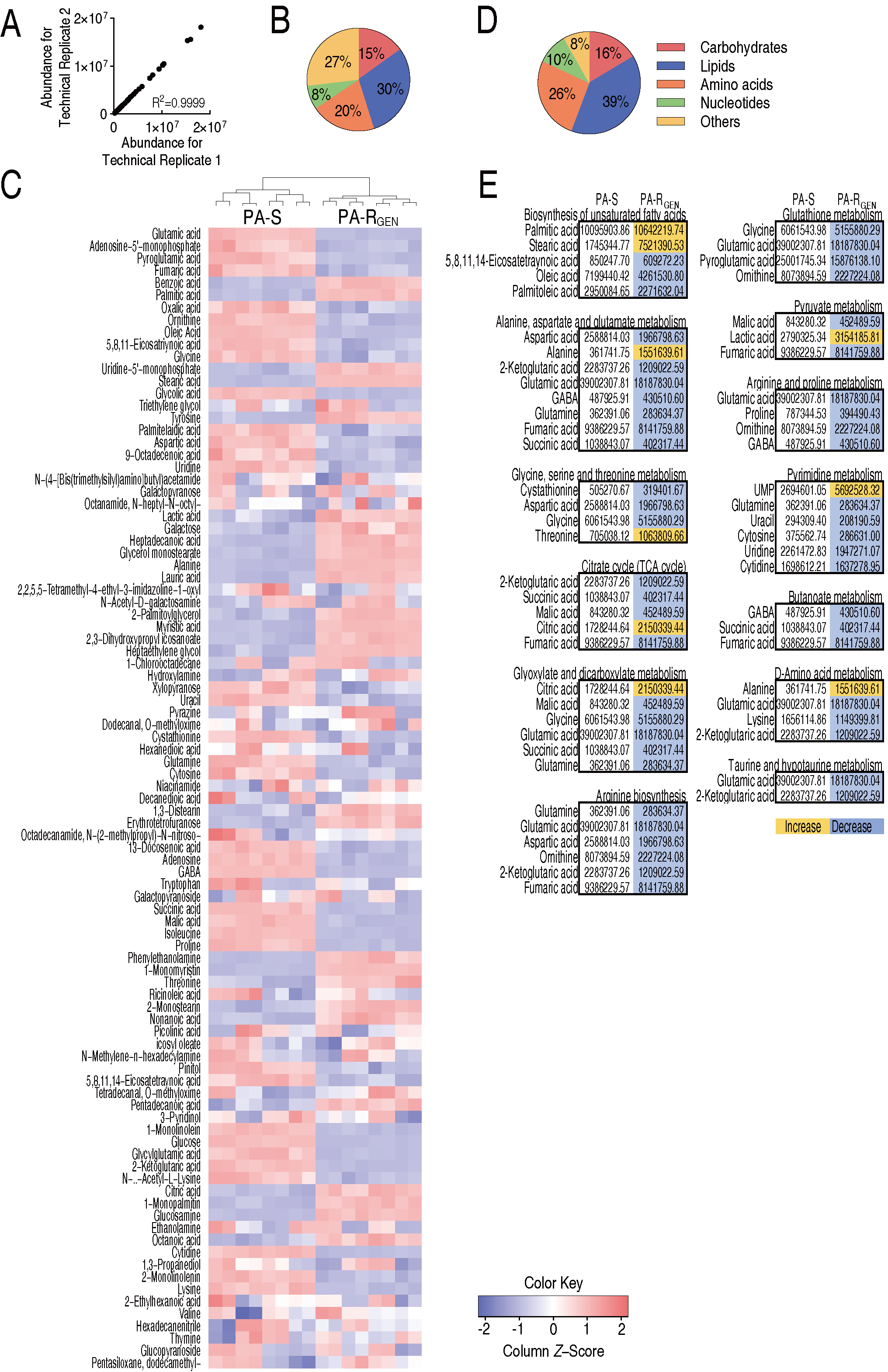


**Supplementary Figure 1 Metabolomic comparison between PA-R_GEN_ and PA-S.** PA-R_GEN_ and PA-S were used to analyze the metabolic changes due to gentamicin resistance. Each strain was set with 4 biological replicates and 2 technical replicates for GC-MS data collection. Metabolomics data were obtained through Agilent Chrom Station software and manually corrected. 93 metabolites with sufficient signal were detected in each baseline. Ribitol was used as an internal standard to normalize the peak area of all metabolites. Among the 93 metabolites, 63 showed abundance differences between PA-S and PA-R_GEN_, calculated using two-sided Mann-Whitney *U* test. (A) The correlation coefficient R^2^ between technical replicates was 0.9999. (B) Classification of 93 identified total metabolites. (C) Global metabolic heatmap. The color change from blue to red represents a change in metabolite abundance from low to high and heat map scale is shown on the bottom. (D) Classification of 63 differential metabolites. (E) Metabolite abundance changes with the color blocks in enriched metabolic pathways.


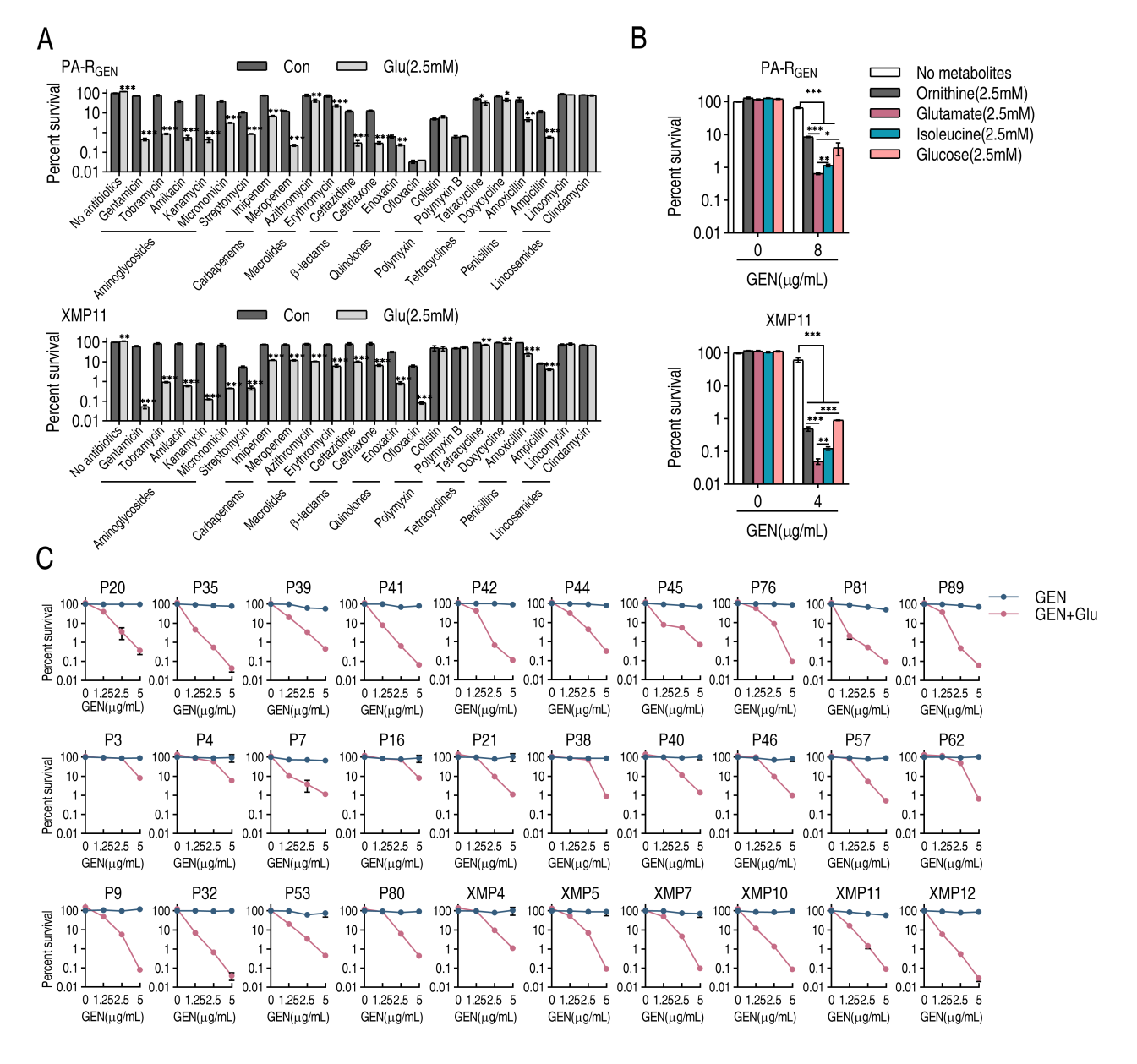


**Supplementary Figure 2 Glutamate-mediated synergy enhances antibiotic killing on clinical isolates.** (A) The synergistic bactericidal effects of 2.5 mM glutamate combined with different antibiotics against PA-R_GEN_ and XMP11 in M9 medium. The various antibiotic doses are shown in Supplementary Table 1. (B) Comparison of the synergistic bactericidal effects of 2.5 mM ornithine, glutamate, isoleucine, and glucose in combination with gentamicin against PA-R_GEN_ and XMP11 in M9 medium. (C) The bactericidal activities of 0, 1.25, 2.5, and 5 μg/mL gentamicin against clinical isolates with or without 2.5 mM glutamate in M9 medium.


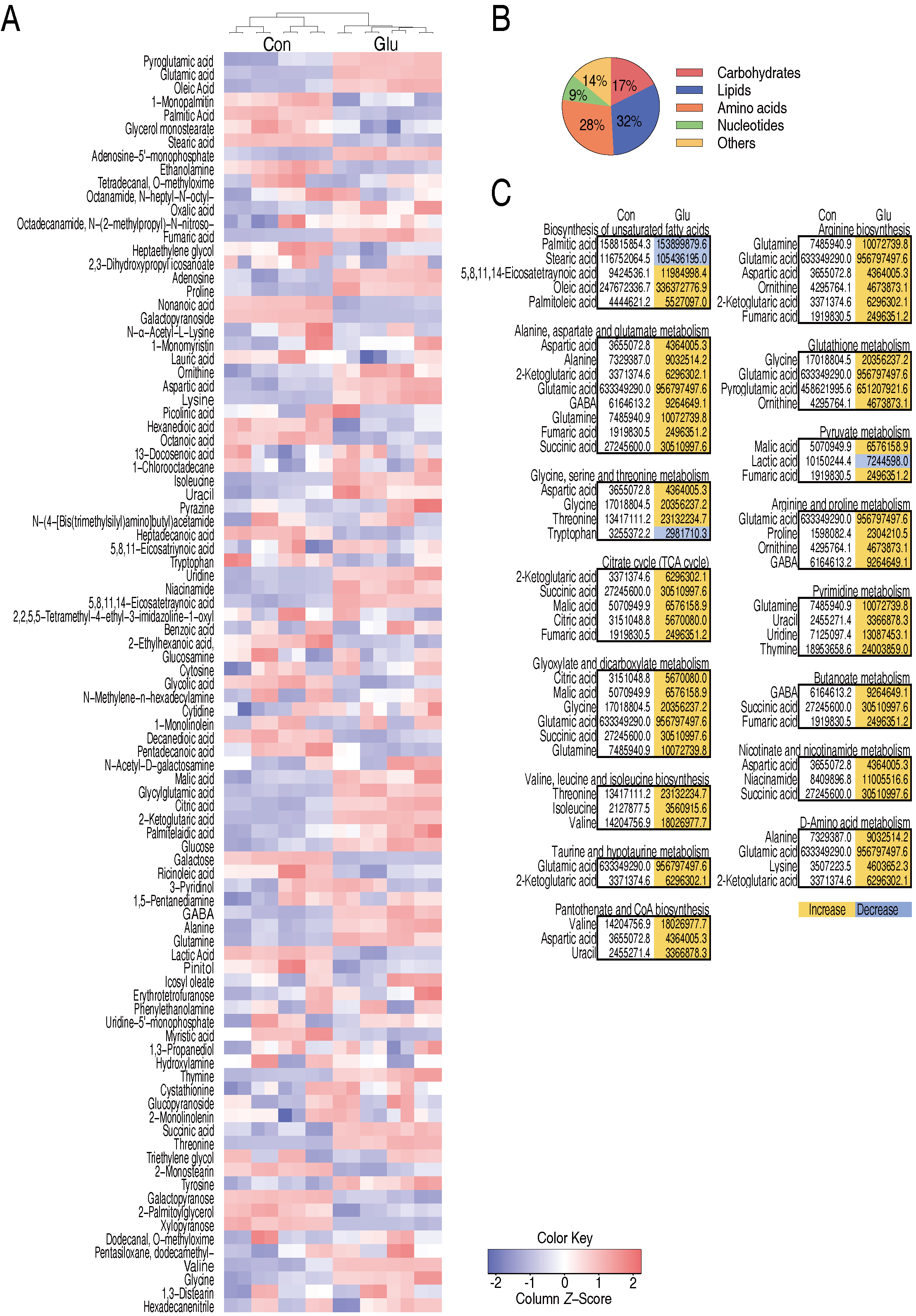


**Supplementary Figure 3 Metabolomic comparison between control group and glutamate group of XMP11.** XMP11 was used to analyze the metabolic changes caused by 2.5 mM glutamate. Control group and glutamate group were set with 4 biological replicates and 2 technical replicates for GC-MS data collection, respectively. Metabolomics data were obtained through Agilent Chrom Station software and manually corrected. 93 metabolites with sufficient signal were detected in each baseline. Ribitol was used as an internal standard to normalize the peak area of all metabolites. Among the 93 metabolites, 57 showed abundance differences between PA-S and PA-R_GEN_, calculated using two-sided Mann-Whitney *U* test. (A) Global metabolic heatmap. The color change from blue to red represents a change in metabolite abundance from low to high and heat map scale is shown on the bottom. (B) Classification of 57 differential metabolites. (C) Metabolite abundance changes with the color blocks in enriched metabolic pathways.

**Supplementary Table 1 The maximum daily dosage of different antibiotics in humans and their corresponding experimental bactericidal concentrations.**

|  | Antibiotics | Maximum daily dosage of different antibiotics in humans (g） | Experimental bactericidal concentrations in PA-R (µg/mL) | Experimental bactericidal concentrations in XMP11 (µg/mL) |
| --- | --- | --- | --- | --- |
| Aminoglycosides | Gentamicin | 0.30 | 10 | 5 |
|  | Tobramycin | 0.30 | 10 | 5 |
|  | Amikacin | 0.90 | 30 | 15 |
|  | Kanamycin | 0.90 | 30 | 15 |
|  | Micronomicin | 0.36 | 12 | 6 |
|  | Streptomycin | 2.00 | 67 | 33 |
| Carbapenems | Imipenem | 1.50 | 50 | 25 |
|  | Meropenem | 1.50 | 50 | 25 |
| Macrolides | Azithromycin | 0.50 | 17 | 8 |
|  | Erythromycin | 1.00 | 33 | 17 |
| β-lactams | Ceftazidime | 4.00 | 133 | 67 |
|  | Ceftriaxone | 4.00 | 133 | 67 |
| Quinolones | Enoxacin | 0.60 | 20 | 10 |
|  | Ofloxacin | 0.75 | 25 | 13 |
| Polymyxin | Colistin | 0.30 | 10 | 5 |
|  | Polymyxin B | 0.12 | 4 | 2 |
| Tetracyclines | Tetracycline | 2.00 | 67 | 33 |
|  | Doxycycline | 0.20 | 7 | 3 |
| Penicillins | Amoxicillin | 1.50 | 50 | 25 |
|  | Ampicillin | 8.00 | 267 | 133 |
| Lincosamides | Lincomycin | 1.20 | 40 | 20 |
|  | Clindamycin | 12.00 | 40 | 20 |

**Supplementary Table 2 MICs of various antibiotics for clinical strains of antibiotic-sensitive *P. aeruginosa*.**

|  | Strain | PB | GEN | MEM | CAZ | LEV | TZP | AZT |
| --- | --- | --- | --- | --- | --- | --- | --- | --- |
| S-PA | S1 | 2 | 2 | 0.5 | 2 | 1 | 4 | 4 |
|  | S9 | 2 | 4 | 1 | 2 | 0.5 | 4 | 4 |
|  | S14 | 2 | 4 | 1 | 2 | 1 | 2 | 4 |
|  | S19 | 2 | 4 | 0.5 | 2 | 1 | 2 | 4 |
|  | S24 | 2 | 2 | 1 | 1 | 1 | 2 | 2 |
|  | S25 | 2 | 4 | 2 | 4 | 0.5 | 8 | 8 |
|  | S26 | 1 | 2 | 1 | 2 | 0.5 | 4 | 4 |
|  | S30 | 2 | 2 | 0.5 | 1 | 1 | 2 | 2 |
|  | S32 | 0.5 | 4 | 1 | 1 | 1 | 2 | 2 |
|  | S34 | 1 | 1 | 1 | 1 | 1 | 1 | 1 |
|  |  |  |  |  |  |  |  |  |
|  |  |  |  |  |  | S | I | R |

**Supplementary Table 3 Primers used for qPCR of the *P.aeruginosa*.**

| gene | primer |
| --- | --- |
| *ref-F* | CAAAACTACTGAGCTAGAGTACG |
| *ref-R* | TAAGATCTCAAGGATCCCAACGGCT |
| *accA-F* | GCTGAACAGGTAGCCGATGT |
| *accA-R* | GCAAGCCAAGATCGAAGAGC |
| *accB-F* | AGCGTGAAGAAAGGCGACAT |
| *accB-R* | AACTTCGGCTTCGATGTGGT |
| *accC-F* | GCCACCAGAAGGTGATCGAA |
| *accC-R* | GTAGCCGATCTCGATGCAGG |
| *accD-F* | GAACCGCCCATGAAGGAGAA |
| *accD-R* | AAGTACAAGGATCGCCTCGC |
| *fabI-F* | AGGTAGGAGAGGGTCAGCAG |
| *fabI-R* | TCAGCGCCTACAGCTTCATC |
| *fabA-F* | TGACAGGCGAAGAACCACAG |
| *fabA-R* | CCAAACAACACGCCTTCACC |
| *fabB-F* | GGACGGATTGAAGCGAATGC |
| *fabB-R* | CGGCAATGACAAAGACACCG |
| *fabD-F* | GGGTCTTGTCGGTCTGGTTC |
| *fabD-R* | ATGTCTGCATCCCTCGCATT |
| *fabG-F* | ACCACCTTGGCGATCTCTTC |
| *fabG-R* | TCATCGACACCGACATGACC |
| *fabZ-F* | CCGCACAGATGATTTCAGCC |
| *fabZ-R* | CTGCACGCCAAGTTCATCAG |
| *fabF1-F* | AACAGGGATCGGCAGTTGTT |
| *fabF1-R* | GACCTGTCCGCCTATTCCAC |
| *fabH2-F* | CAGGCCTCCAACTACTTCCG |
| *fabH2-R* | ATGTTGCTCACCACTCGCTC |
| *desA-F* | *GAAGAGCTGCACAACAACCA* |
| *desA-R* | *GGAAGCGGTTGTTGAGGATC* |
| *desB-F* | *CTGGGCAAGATCCTCGAGAA* |
| *desB-R* | *GATATGGTTGTGGGTGTGCC* |
| ant(2'')-I-F | ATCGTCGTGCATGGTGATGT |
| ant(2'')-I-R | CGAAGATGCCCGCAAGAATG |
| aac(6')-I-F | CAACGTTGCGTTCTTGGAGG |
| aac(6')-I-R | CGTACTTGAGCAACCTCCGT |
| aac(3)-II-F | TTCGTCAAGCAGGAAGTGCT |
| aac(3)-II-R | TCGCTCTGGGTTTCGTTCAG |
| aac(3)-I-F | TTTCGGTCGTGAGTTCGGAG |
| aac(3)-I-R | GCTCAAACTTGGGCAGAACG |
